# Clinical management and burden of bipolar disorder: a multinational longitudinal study (WAVE-bd Study)

**DOI:** 10.1186/1471-244X-11-58

**Published:** 2011-04-11

**Authors:** Eduard Vieta, Elena Blasco-Colmenares, Maria Luisa Figueira, Jens M Langosch, Miriam Moreno-Manzanaro, Esteban Medina

**Affiliations:** 1 Bipolar Disorders Programme, Hospital Clínic, University of Barcelona, IDIBAPS, CIBERSAM, Villarroel 170, 08036 Barcelona, Spain; 2 Welch Center for Prevention, Epidemiology, and Clinical Research. Johns Hopkins Bloomberg School of Public Health, 2024 E. Monument St., Baltimore, MD 21205, USA; 3 Psychiatric Department, Hospital Santa Maria, Faculty of Medicine, University of Lisbon, Av. Prof. Egas Moniz, 1640-035 Lisboa, Portugal; 4 Bethanien Hospital for Psychiatry, Psychosomatics, and Psychotherapy, Gützkower Landstraße 69, 17489 Greifswald, Germany; 5 Medical Department, AstraZeneca Pharmaceuticals, Serrano Galvache 56, 28033 Madrid, Spain

## Abstract

**Background:**

Studies in bipolar disorder (BD) to date are limited in their ability to provide a whole-disease perspective - their scope has generally been confined to a single disease phase and/or a specific treatment. Moreover, most clinical trials have focused on the manic phase of disease, and not on depression, which is associated with the greatest disease burden. There are few longitudinal studies covering both types of patients with BD (I and II) and the whole course of the disease, regardless of patients' symptomatology. Therefore, the Wide AmbispectiVE study of the clinical management and burden of Bipolar Disorder (WAVE-bd) (NCT01062607) aims to provide reliable information on the management of patients with BD in daily clinical practice. It also seeks to determine factors influencing clinical outcomes and resource use in relation to the management of BD.

**Methods:**

WAVE-bd is a multinational, multicentre, non-interventional, longitudinal study. Approximately 3000 patients diagnosed with BD type I or II with at least one mood event in the preceding 12 months were recruited at centres in Austria, Belgium, Brazil, France, Germany, Portugal, Romania, Turkey, Ukraine and Venezuela. Site selection methodology aimed to provide a balanced cross-section of patients cared for by different types of providers of medical aid (e.g. academic hospitals, private practices) in each country. Target recruitment percentages were derived either from scientific publications or from expert panels in each participating country. The minimum follow-up period will be 12 months, with a maximum of 27 months, taking into account the retrospective and the prospective parts of the study. Data on demographics, diagnosis, medical history, clinical management, clinical and functional outcomes (CGI-BP and FAST scales), adherence to treatment (DAI-10 scale and Medication Possession Ratio), quality of life (EQ-5D scale), healthcare resources, and caregiver burden (BAS scale) will be collected. Descriptive analysis with common statistics will be performed.

**Discussion:**

This study will provide detailed descriptions of the management of BD in different countries, particularly in terms of clinical outcomes and resources used. Thus, it should provide psychiatrists with reliable and up-to-date information about those factors associated with different management patterns of BD.

**Trial registration no:**

ClinicalTrials.gov: NCT01062607

## Background

Bipolar disorder (BD) is not just a single disorder, but a category of lifelong mood disorders characterised by the presence of one or more recurrent manic, hypomanic and depressive episodes. Individuals who experience manic episodes also commonly experience depressive episodes or symptoms, or mixed episodes in which features of both mania and depression are present. While these episodes are usually separated by periods of normal mood, in some patients depression and mania may rapidly alternate [1].

Estimates for lifetime prevalence of any type of BD range from 0.5% to 5%. However, caution must be used when comparing studies, as the diagnostic assessment methods and criteria used to formulate diagnoses vary from study to study [2]. A recent review of epidemiological studies, which aimed to determine the prevalence of BD in Europe, revealed a remarkable degree of consistency across diverse study designs and between countries. The lifetime prevalence rate of mania (BD type I) appears to be very similar across studies, with estimates ranging from 0.1-0.2% to 1.8%. There is reasonably consistent evidence that BD-I and BD-II disorders, diagnosed according to criteria in the fourth edition of the Diagnostic and Statistical Manual of Mental Disorders (DSM-IV) [1], have an estimated 1-year prevalence of approximately 1%, with no major differences by age group and gender [3].

Over 90% of patients with BD experience recurrences during their lifetime [4], often within 2 years of the initial episode, and the consequences of recurrent illness are substantial for patients. Most randomised controlled trials investigating the efficacy of guideline-based treatment with current drug therapies, or with new emerging therapies, have assessed recurrence in patients who had initially recovered from a mood episode. A further need is to identify the association between patient characteristics and clinical characteristics as predictors of recurrence. This information may allow clinicians to better understand the course of the disease, and to focus on clinical management of those factors with a significant impact on disease outcomes [5].

The emerging picture of the course of BD is quite heterogeneous and includes slow or incomplete recovery from acute episodes, continued risk of recurrences, and sustained morbidity over time, even with continuous long-term use of current treatments. Recovery from an acute episode of mania, even if treatment is established very early in the course of the disorder, may require 3-6 months and thus may no longer meet the standard diagnostic criteria for an acute episode (syndromal remission). Achieving symptomatic remission, defined as the presence of minimal symptoms, may take longer and an 2 additional months may be needed to attain the start of recovery, defined as a sustained remission. Time to remission is even longer following repeated recurrences of BD [6].

Moreover, BD can adversely affect the individual, reducing health-related quality of life and functioning, including employment and productivity at work [7]. It is becoming increasingly recognised that BD is associated with a higher level of functional impairment than previously thought, particularly with regard to social adjustment and vocational functioning [6,7].

In addition to patient burden, caregiver burden is currently one of the key factors in managing patients with BD. The term "caregiver burden" refers to the emotional, social, and financial stresses that caring for a relative or friend with mental illness imposes on the caregiver, and is defined as "the presence of problems, difficulties or adverse events which affect the life of psychiatric patient's caregivers" [8]. On the basis of the method established by Pollak and Perlik [9] the primary caregiver is defined as the family member, friend or significant other who satisfied the greatest number (and at least three) of five criteria, namely: a spouse, parent or spouse equivalent; has the most frequent contact with the patient; helps to support the patient financially; has been the most frequent collateral participant in the patient's treatment; and is the person contacted by treatment staff in case of emergency.

While caregivers can accept some of the burden for the care of patients with BD, management of the disease also places a substantial burden on healthcare providers. BD typically places greater demand on hospital psychiatric services than non-BD depression [10]. A study that permits comparison of different healthcare practices between countries may help to optimise resource utilisation.

One of the key challenges in ensuring that a new study produces meaningful data is to avoid any selection bias in recruitment of both investigators and patients. This can be achieved by having as few exclusion criteria as possible. A further challenge is to recruit patients without contravening the diverse range of laws, which vary within and between countries, covering privacy relating to the acquisition and use of medical data. A recent study in another area of medicine has attempted to avoid investigator bias in patient selection by engaging a third party to manage patient selection and recruitment [11]. In that study, patients were able to opt out without consulting their physician.

In view of the above, the overall objectives of the Wide AmbispectiVE study of the clinical management and burden of Bipolar Disorder (WAVE-bd) study are: to provide accurate and reliable information on the management of patients with BD in conditions representative of everyday clinical practice; to determine the clinical outcomes of such management and the use of resources in relation to the disease; and to establish the factors associated with different management patterns and clinical and functional outcomes.

## Methods

### Study design

**Figure 1:**
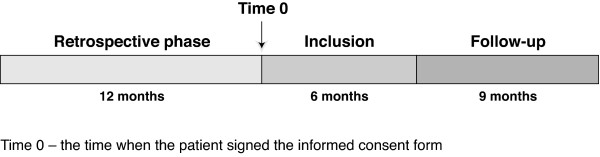
**Study design**.

The WAVE-bd study (NCT01062607) is a multinational, multicentre, observational, longitudinal or cohort study of patients diagnosed with BD type I or II with at least one mood event in the 12 months prior to the study start (Time 0, Figure 1).

The study comprises two different follow-up phases; one retrospective and one prospective (ambispective design). The retrospective phase for each patient started from the index event, which occurred a maximum of 12 months and minimum of 3 months before Time 0, and ended when the patient signed the informed consent form. Information from medical records related to the patient and their disease during that period (i.e. retrospective information - from index event to inclusion) was recorded in the electronic case report form (eCRF) at the inclusion visit. The prospective phase started when the first patient signed the informed consent form and will end when the last patient included in the study attends their final visit. Data for prospective analysis are collected as described at all visits (including the inclusion visit). All throughout the prospective phase, the required information will be recorded in the eCRF and the questionnaires completed every time the patient attends the psychiatrist's office (unless otherwise stated). The psychiatrist will schedule visits according to real-life clinical practice. No interventions, extra procedures, or extra visits will be required for the purpose of the study.

The study design means that patients will spend variable amounts of time in the study depending on the date of signing the informed consent form, the length of the enrolment period, and the date of their index episode. The minimum time in the study is 12 months and the maximum 27 months, considering the retrospective and prospective phases, and the 6 month enrolment period together (Figure 1).

### Study population

One aim of the study is to determine how patients with BD are managed in different settings and countries (Austria, Belgium, Brazil, France, Germany, Portugal, Romania, Turkey, Ukraine and Venezuela). It is therefore important to obtain a patient population that is representative of real-world practice. The inclusion of sites and patients was determined on that basis.

### Sites and investigators

Since the type of site is a variable that might influence the management of patients with BD and the profile of patients attending each type of site may be different, it was necessary to select different types of sites to obtain a representative sample. Generally, patients with BD are seen in mental health centres, clinics, private settings, hospitals or specialised units. Since this distribution varies from one country to another, the study centre selection process had to be adapted locally.

Selection of participating sites was based on the percentage of patients that attend different types of sites in each country, thus ensuring that patients attending one specific site type are not over-represented in the study sample (Table 1). These percentages were obtained either from the literature or from expert panels from each participating country.

**Table 1:** Patient recruitment by country and type of study centre

Country	Site type	Patients per site type (%)	Total patients enrolled
Austria	University hospital	16	20
	Private practice	65	81
	General hospital	19	24

Belgium	Clinics	46	190
	Private practice	31	129
	General practitioner	23	95

Brazil	Public hospital	88	146
	University hospital	12	20

France	Hospital/clinics	49	247
	Private practice	51	260

Germany	University hospital	27	59
	Community hospital	21	46
	Private practice	52	114

Portugal	Hospital	24	123
	Private practice	57	295
	Mental health clinic	19	98

Romania	Ambulatory	37	68
	Hospitals	63	115

Turkey	Private practice	39	151
	University hospital	42	162
	State hospital	18	70

Ukraine	Mental health clinic	90	199
	Private practice	10	22

Venezuela	Private practice	33	76
	Public hospital	67	155

The number of participating investigators is sufficient to ensure: 1) that there is a representative sample of the whole psychiatrist population in each country; and 2) inclusion of a sufficient number of patients to provide the required sample size calculated for the study. No other criteria were applied to selection and inclusion of investigators, in order to avoid any selection bias.

Approximately 250 study centres in 10 countries are participating in the study. Based on the criteria described above, target percentages of patients recruited by each type of study centre were devised to reflect management practices in each of the participating countries (Table 1). The study data will be able to provide a global perspective on comparisons between public *vs *private care, academic hospital *vs *standard hospital care, and hospital *vs *non-hospital care.

### Patient population

Each investigator identified all patients with at least one mood event in the 12 months prior to the beginning of the study (Time 0), except for those whose index mood event occurred in the 3 months before the study start.

The aim of the study is to achieve systematic inclusion of patients, therefore, where possible, every eligible patient identified at each of the study centres was invited to participate. The main exceptions were at those sites that saw a high number of eligible patients during the recruitment period. At these sites, investigators were allowed to recruit a representative sample using an electronic application, which makes selections by simple randomisation, in order to avoid any type of selection bias.

Patients aged ≥ 18 years, with a diagnosis of BD type I or II (DSM-IV-TR [1]) in any phase of the disorder and who had at least one mood event (depression, mania, hypomania or mixed) according to the DSM-IV-TR definitions during the 12 months prior to the beginning of the study were eligible for recruitment, except for those starting the index mood event less than 3 months before Time 0, subject to their providing informed consent. However, those patients starting the index mood event more than 3-months before Time 0 were eligible even if the index event was not resolved, or if the index event had resolved and they subsequently initiated another event.

Patients not eligible to participate in the study were those participating in an interventional clinical study and any patients unable to complete Patient Reported Outcomes questionnaires.

### Measurements

The WAVE-bd study aims to provide a wide-ranging picture of BD management practices, and impact on patients, caregivers and healthcare resource use across a range of countries. For this reason, several measuring instruments are being employed to maximise the types of data collected. All instruments have been validated before the study start, including linguistic validation where needed. In all cases, the scales were also psychometrically validated, at least in the original language. Information regarding demographics (sex, race, age, educational level, professional status, degree of disability, degree of independence or co-residence), alcohol and other substance abuse, medical history, disease characteristics (date of first diagnosis, type of BD, family history of psychiatric diseases, episodes during the last 12 months, presence of psychotic symptoms, hospital admissions, and suicide attempts), treatments received (drug, schedule and dose, and whether the patient received psychologist or group therapy), healthcare resources use, and clinical outcomes will be collected throughout the study (Table 2).

**Table 2:** Study plan and assessments

Time	First visit*	Each other visit	Last visit
Site information	X		
Patient demographics	X		
Medical history	X		
Disease characteristics	X		
Treatment information	X	X	X
Healthcare resources consumption	X	X	X
Clinical outcomes	X	X	X
Adherence to treatment	X	X	X
Quality of life	X	X	X
Functioning	X	X	X
Caregiver demographics	X**		
Caregiver burden	X**		

* First visit: the visit when the patient signs the informed consent form.

** If the caregiver does not attend this visit, the information will be collected at the next visit that the caregiver attends. This information will be collected (if the caregiver consents) only once during the study period for each caregiver. In the event that the caregiver does not attend any visits, this information will not be collected.

An electronic adaptation of the National Institute of Mental Health prospective Life Chart Methodology (NIMH-LCM™) will be used specifically for assessing clinical outcomes during both the retrospective and the prospective phases of the study. It allows the daily assessment of mood and episode severity, based on the degree of mood-associated functional impairment. The NIMH-LCM provides a visual method of tracking the patient's mood at each visit. Each form covers a 1-month period, and clinicians rate both mania and depression on the chart, list the medications taken by the patient during the month, and record any other non-mood symptoms (prospective phase only), if applicable [12].

During the prospective phase of the study the following assessments will be performed to evaluate clinical outcomes, adherence to treatment, quality of life, patient functioning and caregiver burden:

*CGI-BP scale: *this is an adaptation of the Clinical Global Impressions (CGI) scale designed to assess global illness severity and change in patients with BD. It contains nine items [13].

*DAI-10 scale: *the Drug Attitude Inventory (DAI) is a self-applied scale to measure subjective responses to medication. This instrument reveals whether the patient is satisfied with their treatment and evaluates their understanding of how the treatment is affecting them. The reduced version 'DAI-10' has ten highly specific items of subjective experience. These are based on the true recorded and transcribed accounts of patients, and response options are true/false only. These items were selected for their capacity to discriminate between medication adherence grades in a way that can be analysed statistically. Although the DAI is specific for schizophrenia, it has also been used to investigate treatment adherence in patients with BD [14].

*MPR: *the Medication Possession Ratio (MPR), first described by Sclar *et al *[15], is a formula used to determine adherence measured from the first to the last prescription, with the denominator being the duration from index to the exhaustion of the last prescription and the numerator being the days supplied over that period from first to last prescription. The MPR will also be used for the retrospective phase of the study.

*EQ-5D questionnaire: *this is a standardised instrument used to measure health outcomes [16]. It is applicable to a wide range of health conditions and treatments, and it provides a simple descriptive profile and a single index value for health status. The respondent is asked to indicate their health state by marking the box against the most appropriate statement in each of the five dimensions. This decision results in a one-digit number expressing the level selected for each dimension. The digits for five dimensions can be combined in a five-digit number describing the respondent's health state. It should be noted that the numerals 1-3 have no arithmetic properties and should not be used as a cardinal score.

*FAST scale: *the Functioning Assessment Short Test (FAST) is a brief instrument designed to assess the main functional problems experienced by psychiatric patients, particularly bipolar patients. It comprises 24 items that assess impairment or disability in six specific function domains: autonomy, occupational functioning, cognitive functioning, financial issues, interpersonal relationships and leisure time. The total score across all domains will be measured [17].

*BAS: *The Burden Assessment Scale *(*BAS) was developed by Reinhard and Horwitz [18] and will be assessed once during the study. The questionnaire contains 19 items that capture both objective and subjective consequences of providing ongoing care to the seriously mentally ill. The scale distinguishes burden from the measurement of the ill relative's disruptive behaviours and the family's care-giving activities. These are viewed as predictors rather than aspects of burden [18].

### Statistical analyses

Descriptive statistics will include frequency tables (n, mean, median, standard deviation, minimum and maximum for continuous variables and n, frequency and percentage for categorical values). For the population estimation of the variables, the two-sided 95% confidence interval will be obtained.

In order to assess the association of patient characteristics (including functioning and quality of life status) and clinical management (an independent variable) with clinical outcome variables (clinical evolution, mood events, treatment-related events, suicide attempts, variation in scales), logistic and general linear models have been planned. Interest will focus separately on the management differences between the models or groups of models. Model-based point estimates of odds ratios and corresponding 95% confidence intervals will be reported. P-values will be reported for the comparison between different treatments. Since visit-by-visit information from the study index event is being collected, there will be data on patient status during the whole study period. Therefore, it will be possible to analyse data, relative to the time of mood event initiation.

Cox models for survival outcomes, and mixed models for longitudinal data using country as indicator variables, adjusted for age and gender, have been planned in order to investigate differences in clinical outcomes and related factors between countries. A descriptive analysis has been planned to assess factors related to consumption of healthcare resources and caregiver burden, according to caregiver-reported outcomes.

### Sample size

The sample size was calculated in order to ensure that the study obtains meaningful data for descriptive purposes of general clinical management and clinical outcomes at a country level. The main outcome used in the sample size calculation was the proportion of episodes in 1.5 years and the goal was to estimate this proportion in the study population. The minimum number of patients required was estimated, based on an expected proportion of patients with episodes in one year of 35%, assuming an alpha = 0.05, power = 0.80 and a precision of 0.05 (with a 95% confidence interval). The estimated sample size was 370 patients per country (or 400 if it is assumed that approximately 10% will be lost to follow up). Fewer patients per country would provide information on the proportion of episodes with a precision less 5%. It was estimated that approximately 3,200 patients across different countries would be included in the study, but that this number might vary depending on the number of participating countries and sample size in each.

### Safety

The non-interventional nature of this study means that safety data will not be collected proactively. However, spontaneously reported safety events will be communicated to the appropriate health authority, as required by post-marketing pharmacovigilance regulations.

### Study ethics and patient confidentiality

The study will be performed in accordance with ethical principles that are consistent with the Declaration of Helsinki 2008 revision of the International Conference on Harmonisation - Good Clinical Practice (ICH GCP) guidelines and the applicable legislation on non-interventional studies.

The study protocol and informed consent form were approved in writing by the relevant ethics committees in each participating country, as follows: Austria; EC of Salzburg: Belgium; Comité d'Ethique, CUB Hôpital Erasme; Ethische Commissie, vzw Gezondheidszorg Oostkust; Comité d'Ethique, Hôpital Saint Joseph; Comité d'Ethique Hospitalier/HPBV, Hôpital Psychiatrique du Beau Vallon; Comité d'Ethique OM045, Clinique St.-Pierre; Comité d'Ethique Hospitalier-Facultaire Universitaire de Liège, Centre Hospitalier Universitaire du Sart Tilman; Ethische Commissie/Coördinator klinische studies, AZ Sint-Lucas Brugge; Commissie voor Medische Ethiek, Psychiatrisch Zkh. Onze Lieve Vrouw; Comité d'Ethique, Cliniques Universitaires UCL de Mont-Godinne; Commissie Medische Ethiek - Toetsingscommissie, Campus Gasthuisberg; Comité d'Ethique, U.C.L. - Faculté de Médecine; Commissie voor Ethiek, AZ St.-Jan Brugge; Comité d'Ethique, C.H.P. Petit Bourgogne; Toetsingscommissie Ethiek GGZ Broeders van Liefde, U.P.C. Sint-Kamillus; Comité d'Ethique, C.H.P. Les Marronniers; Secrétariat du Comité d'Ethique ISPPC, CHU A. Vésale; Comité d'Ethique, CUB Hôpital Erasme; Comité d'Ethique, Vivalia Centre Universitaire Provincial La Clairière: Brazil; Comissão Nacional de Ética em Pesquisa; Comitê de Ética em Pesquisa da Maternidade Climério de Oliveira; Comitê de Ética em Pesquisa do Hospital Irmãos Penteado - Irmandade de Misericórdia de Campinas; Comissão de Ética para Análise de Projetos de Pesquisa - CAPPesq da Diretoria Clínica do Hospital das Clínicas e da Faculdade de Medicina da USP; Comitê de Ética em Pesquisa da Faculdade de Medicina de Botucatu; Comitê de Ética em Pesquisa da Universidade Federal de Goiás (CEPHMA/HC/UFG); Comitê de Ética em Pesquisa do Hospital Pró-Cardíaco; Comitê de Ética em Pesquisa da Secretaria de Estado da Saúde de Santa Catarina - CEP-SES/SC; Comite de Ética em Pesquisa da Faculdade de Medicina do ABC: France: IEC of of Ile de France VI; Doctors Governing Body: Germany; Ethikkommission.Ernst-Moritz Arndt Universität Greifswald: Portugal; Data Privacy Authority (Comissão Nacional de Protecção de Dados); Comissão de Ética para a Saúde; Comissão de Ética do Hospital dos Lusíadas; Comissão de Ética da Clínica Psiquiátrica de S. José; Comissão de Ética do Hospital de Magalhães Lemos, EPE; Comissão de Ética da Saúde do Hospital de São João; Comissão de Ética do Hospital Infante D. Pedro EPE; Comissão de Ética do Centro Hospitalar Médio Tejo, EPE; Comissão de Ética do Centro Hospitalar de Setúbal, EPE: Romania; National Drug & Medical Devices Agency; National Ethics Committee: Turkey; Central IRB within MoH: Ukraine; Central Ethics Committee of Ministry of Health of Ukraine: Venezuela; RA: Instituto Nacional de Higiene "Rafael Rangel"; Comité de Bioética Hospital Universitario de Caracas; Centro Nacional de Bioética de Venezuela; Instituto Autónomo Hospital Universitario de Los Andes; Comisión de Ética del Hospital Vargas.

Investigators will perform the study in accordance with the regulations and guidelines governing medical practice and ethics in their country and in accordance with currently acceptable techniques. Patients and caregivers will authorise the collection, use and disclosure of their personal data by the investigator and by those persons who need that information for the purposes of the study. Study data will be stored in a computer database, maintaining confidentiality in accordance with local laws for data protection.

## Discussion and conclusions

Current treatment guidelines for BD are based on the results of published randomised clinical trials and meta-analyses. Although these methods are the most reliable sources of evidence, they also have limitations arising from restricted study populations. Many studies may lack external validity due to strict inclusion criteria, which usually do not allow the inclusion of patients with severe disease or those with co-morbidities such as alcohol and drug dependence, or anxiety disorders that are highly prevalent among patients with BD.

Very few longitudinal observational studies carried out to date have studied BD from a comprehensive perspective, i.e. providing a global perspective of a representative sample of patients and simultaneously considering all phases of the disease. Most have focused on only one of the phases, either manic or depressive, or have researched patients who were attending tertiary settings only or receiving certain specific treatments [19-22].

The complex nature of bipolar illness may complicate the measurement of impairment. In addition to other potential determinants of functional impairment in BD, cognitive impairment may be an important factor. Most patients with BD consistently show evidence of impairments in executive functioning, attention, verbal and working memory, and tests of visuospatial function. Recent studies in currently euthymic patients with BD suggest that such deficits can persist even after apparent clinical recovery, and so cannot be entirely ascribed to acute, state-related cognitive deficits that are well known to occur in acute episodes of emotional disease [6]. The impact of these cognitive deficits on functioning is remarkable [23,24].

To date, few studies have investigated the care-giving stresses and the associated health and mental health risks among the family and friends of patients with BD [25-27]. Results from these studies reveal that up to 93% of caregivers of bipolar patients suffer from a moderate or higher level of stress when the patient is admitted to an inpatient unit or outpatient clinic; moreover, 70% of caregivers still report a moderate-to-high burden 15 months after patient admission. Higher levels of caregiver burden at the time of patient admission were associated with increased depression and use of mental health services by caregivers during the previous 7 months. Poorer social and occupational functioning, an episode in the last 2 years, history of rapid cycling, and the caregiver being responsible for medication intake explained a quarter of the variance of the caregiver's subjective burden in a landmark study [28].

The large, non-interventional WAVE-bd study is expected to provide a comprehensive comparison of the routine management of patients with BD across different countries, particularly in terms of clinical outcomes and resources used, and to determine the consequences of such management. A key consideration is whether the study population is truly representative of the overall population of patients with BD. Inclusion of patients was based on random selection from the whole BD population recorded by each investigator. This methodology, added to the large sample size, ensures that the number of patients of each type is representative of the actual population diagnosed in real life.

Since WAVE-bd is a multinational study, it is possible that application of diagnostic criteria will vary slightly between regions and countries and some patients, especially those with type II BD who could have been included, will be given a different diagnosis and managed for major depressive disorder or even schizophrenia, rather than BD. However, inclusion of misdiagnosed patients would introduce bias, since the main objective of this study is to evaluate treatment practices for BD.

All of the studies described above were based on low rates of recruitment from the eligible population. A total of 3188 randomly selected patients were invited to participate in the WAVE-bd study. Of these, 2965 (93%) accepted the invitation to participate and provided written informed consent. The other 7% of patients screened declined to participate and will not be included in the analysis. The systematic approach to inclusion of patients in the WAVE-bd study and stratification by type of study centre to reflect local practice, combined with the 93% patient acceptance rate, leads us to believe that the study population avoids selection bias and is truly representative of the overall global population with unstable BD. Consequently, the investigators believe that the study promises to provide psychiatrists with reliable and up-to-date information about the factors associated with different management patterns of BD on a global scale.

## Competing interests

EV, MLF and JML have received reimbursements, fees, and funding from AstraZeneca for participating in clinical studies and advisory boards. MMM and EM are employees of AstraZeneca at the time of submitting this manuscript. The study is funded by AstraZeneca; Clinical Trials Registry number: NCT01062607.

## Authors' contributions

EV, MLF, JML, EBC and EM conceived the study, participated in its design and performed the statistical analysis. MMM participated in the study design and coordination and helped to draft the manuscript. All authors read and approved the final manuscript.

## Pre-publication history

The pre-publication history for this paper can be accessed here:

http://www.biomedcentral.com/1471-244X/11/58/prepub
